# Biomechanics of Third Window Syndrome

**DOI:** 10.3389/fneur.2020.00891

**Published:** 2020-08-25

**Authors:** Marta M. Iversen, Richard D. Rabbitt

**Affiliations:** ^1^Department of Biomedical Engineering, University of Utah, Salt Lake City, UT, United States; ^2^Department of Otolaryngology, University of Utah, Salt Lake City, UT, United States; ^3^Neuroscience Program, University of Utah, Salt Lake City, UT, United States

**Keywords:** biomechanics, canal dehiscence, superior semicircular canal dehiscence, third window, vestibular, dizziness, vertigo, air-bone gap

## Abstract

Third window syndrome describes a set of vestibular and auditory symptoms that arise when a pathological third mobile window is present in the bony labyrinth of the inner ear. The pathological mobile window (or windows) adds to the oval and round windows, disrupting normal auditory and vestibular function by altering biomechanics of the inner ear. The most commonly occurring third window syndrome arises from superior semicircular canal dehiscence (SSCD), where a section of bone overlying the superior semicircular canal is absent or thinned (near-dehiscence). The presentation of SSCD syndrome is well characterized by clinical audiological and vestibular tests. In this review, we describe how the third compliant window introduced by a SSCD alters the biomechanics of the inner ear and thereby leads to vestibular and auditory symptoms. Understanding the biomechanical origins of SSCD further provides insight into other third window syndromes and the potential of restoring function or reducing symptoms through surgical repair.

## Introduction

The fluid-filled inner ear is almost completely encased in rigid bone, with the exception of a few compliant windows connecting to the middle ear or cranial cavity. The primary and secondary windows are the oval and round windows, which are responsible for sound transmission from the middle ear to the cochlea. The lymph fluids filling the bony labyrinth are nearly incompressible such that, under normal conditions, inward volume velocity at the oval window is accompanied by an equal outward volume velocity at the round window. This fluid flow between the oval and round windows generates a pressure gradient across the cochlear partition that results in a propagating wave toward the apex of the cochlea, activation of cochlear hair cells, and perception of sound (1). Other normal windows of the inner ear include the vestibular aqueduct, cochlear aqueduct, and foramina for blood vessels (2–4), but these windows normally have very high mechanical impedance, owing to their small diameter and long length, and behave mechanically almost as if sealed (5). An enlarged physiologic window (i.e., enlarged vestibular or cochlear aqueduct) or an additional bony dehiscence can create a pathological third window. If sufficiently large, a third window will introduce a low mechanical impedance, thus shunting part of the inner ear fluid pressure and fluid volume flow at the site of the window. The introduction of a compliant third window can have a profound impact on both auditory and vestibular function.

Tullio studied pathologic third window syndrome in the early 20th century, primarily using the pigeon as the animal model. He opened a third window in the semicircular canal bony duct and demonstrated sound-induced eye movements (6). Sound-evoked vertigo or nystagmus are now termed “Tullio phenomenon,” often exhibited as a symptom of third window syndrome. Third window syndrome was first seen in humans with congenital syphilis in the early 20th century who presented with gummatous osteomyelitis and labyrinthine fistulae (7). Hennebert's studies of these patients described eye movements evoked by pressure changes in the external auditory canal, a phenomenon now termed “Hennebert's sign” (8). Since these studies, various causes of the Tullio phenomenon and Hennebert's sign have been reported, such as perilymphatic fistula (9, 10), Ménière's disease (11), and cholesteatoma (12). However, the most common cause is superior semicircular canal dehiscence.

Superior semicircular canal dehiscence (SSCD) in humans was first described by Minor and colleagues in 1998 (13). High-resolution computed-tomography images of the temporal bone revealed dehiscence of the bone above the superior semicircular canal, and imaging was considered the gold standard for diagnosis for a number of years. However, a high rate of false-positive on CT imaging (14–19) motivates the use of physiological indicators of SSCD prior to CT imaging (20), with the most common tests described in subsequent sections. Under current guidelines, patients must present with at least one audiovestibular symptom for a formal diagnosis (21). Symptoms include vestibular indications such as eye movements or dizziness evoked by sound or middle ear/intracranial pressure changes, chronic disequilibrium, oscillopsia; and auditory indications such as autophony, hyperacusis for bone-conducted sounds, conductive hearing loss, and tinnitus. Patients with SSCD can exhibit a variety of these symptoms, though the majority experience some vestibular symptoms (22). Some factors accounting for subject-specific diversity in the array of vestibular and auditory manifestations have been identified, but in most cases, the details are unknown.

A cadaveric survey of 1,000 temporal bones found 0.5% had complete dehiscence, and another 1.4% had significant thinning of bone overlying the superior canal (23). However, clinical presentation of symptoms is less common than anatomic data suggests. Dehiscences vary in size, where even a tiny dehiscence can make vestibular neurons responsive to sound and vibration (24), while a large dehiscence can undergo autoplugging by the dura that dampens lymph motions and superior canal responses (25). Dehiscence can also be complete, or nearly complete (very thin bone), and this likely explains some of the diversity of clinical presentations with SSCD (25).

Other instances of third window syndrome include dehiscence in the posterior or lateral canal and present with clinical symptoms similar to SSCD, though their etiologies can be different (26). The clinical presentation is not specific to the site of a bony defect, and a high-resolution CT is necessary to establish the exact site of dehiscence (20). Other origins include perilymphatic fistula, enlargement of inner ear windows such as the vestibular aqueduct, cochlea-facial nerve dehiscence, and otosclerosis of the internal auditory canal (9, 20, 27–30).

Several studies examining the biomechanical underpinnings of pathologic third window syndrome are useful when interpreting clinical tests and diverse symptoms experienced by SSCD patients (31–34). In this report, we briefly describe clinical audiologic and vestibular tests, and review the biomechanical origins of the third window syndrome.

## Discussion

### Auditory

#### Audiometry

**Figure 1:**
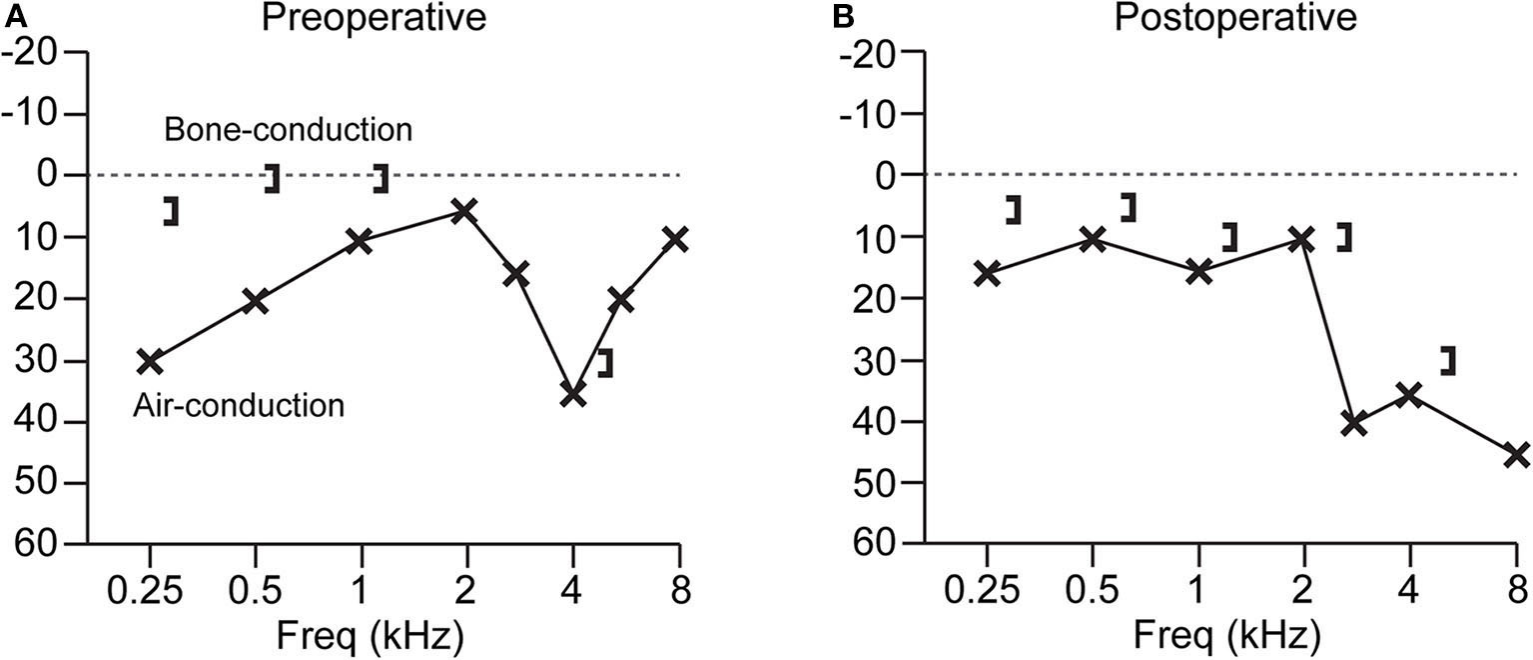
Audiogram from a patient with SSCD before and after superior canal plugging surgery. **(A)** Preoperative audiogram has a low-frequency air-bone gap of up to 25 dB. **(B)** Postoperative audiogram shows resolution of the air-bone gap, with a high-frequency sensorineural loss at 8 kHz. Patient experienced resolution of vestibular symptoms after surgery (41).

Patients with SSCD typically present with an air-bone gap that is largest at low frequencies. There is usually no gap or only a small gap at frequencies >2,000 Hz. Bone conduction thresholds for frequencies <2,000 Hz are sometimes supranormal (0 to −20 dB or more) (35–40). Figure 1 shows an example audiogram with a 25 dB air-bone gap that resolves after canal plugging (41). It is important to properly calibrate audiometers in order to capture possible bone conduction thresholds below 0 dB hearing level (21). Though audiograms and symptoms vary, there is no significant difference in the air-bone gap between patients with vestibular symptoms and those with exclusively auditory symptoms (22).

Other third window conditions have been shown to present with an air-bone gap on audiometry without middle ear pathological findings including: enlarged vestibular aqueduct (42), posterior canal dehiscence (43–45), carotid canal dehiscence on the scala vestibuli side of the cochlea (46), and Paget disease causing microfractures on the scala vestibuli side of the cochlea (40). An air-bone gap is the most common auditory indicator across different third window syndromes. Presence of a third window also alters the acoustic input impedance of the ear, most easily observed at low frequencies (<600 Hz) by measuring motion of the umbo using laser doppler vibrometry or measuring the acoustic power reflectance in the ear canal (47, 48).

#### Auditory Biomechanics

SSCD results in conductive hearing loss by the dual mechanism of worsening air-conduction thresholds and improving bone-conduction thresholds. In normal air-conduction, sound enters the oval window through motion of the stapes and exits the round window with equal and outward motion at the round window membrane. The pressure difference across the cochlear partition drives the traveling wave and sensory hair bundle deflection required for sound perception. When a third window lesion is present on the vestibular side of the cochlear partition (SSCD, enlarged vestibular aqueduct, etc.), acoustic energy is shunted away from the cochlea, primarily at low frequencies, and results in lowered sensitivity to air-conducted sound. In bone conduction, vibration of the inner ear lymph fluids evokes a pressure difference across the cochlear partition that is sensitive to the relative impedance difference between the oval and round windows. When a third window lesion is present on the vestibular side, the impedance difference increases, which putatively is responsible for increased sensitivity to bone-conducted sound (49) and autophony experienced by some patients.

**Figure 2:**
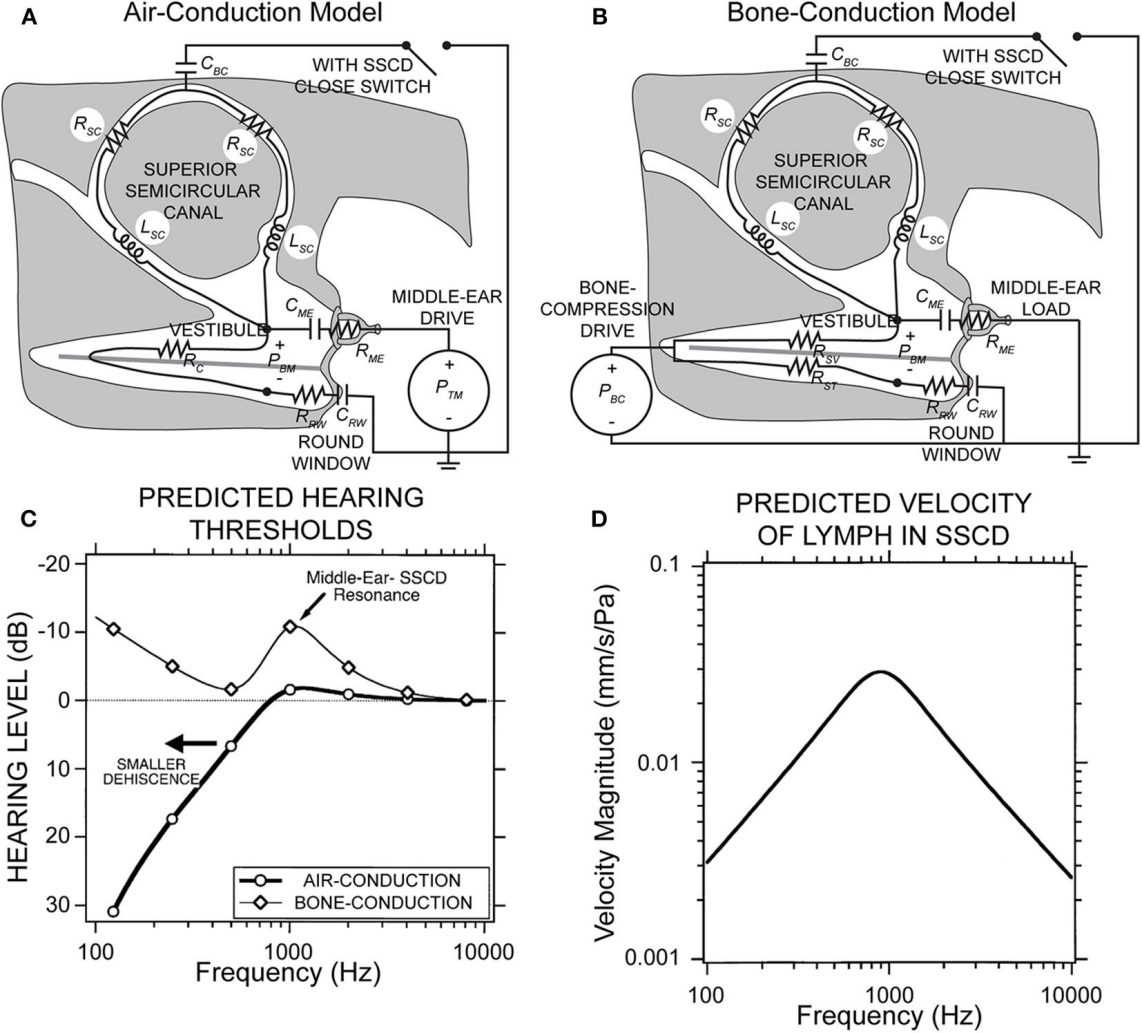
Lumped parameter network model of the inner and middle ear with and without SSCD. **(A)** Air-conduction model where the drive is sound pressure from the ear canal, *P*_*TM*_. **(B)** Bone-conduction model where the drive is effective sound pressure of the vibratory bone-conducted stimulus, *P*_*BC*_. **(C)** The peak in the bone-conduction thresholds is due to a parallel resonance between the compliance of the middle ear load and the inertance of the fluid in the canal limbs. A smaller dehiscence would shift both curves left to lower frequencies. **(D)** Predicted velocity of vestibular lymph fluids in an SSCD with air-conducted sound. Republished from (49), with permission. The Creative Commons license does not apply to this content. Use of the material in any format is prohibited without written permission from the publisher, Wolters Kluwer Health, Inc. Please contact permissions@lww.com for further information.

Figure 2 shows a simplified lumped parameter network model of the inner and middle ear that models air-conducted and bone-conducted sound transmission with and without a SSCD (49). The model is designed for low frequencies (<4,000 Hz) where the wavelengths are longer than the dimensions of inner ear structures. Further, it neglects deformation of membranous labyrinth as well as the cochlear traveling wave. Canal fluid branches were modeled using a resistor and an inductor to describe fluid viscosity and inertia, respectively. The SSCD is modeled as a compliant window (capacitance) which allows pressure relief and volume velocity through the canal branches of the model. Sound pressure across the basilar membrane is analogous to voltage across the cochlear partition and is used to estimate hearing function. The air-conducted sound audiogram predicted by this model exhibits low-frequency hearing loss due to the impedance through the SSCD, which shunts acoustic energy away from the cochlea (Figure 2C). The corner frequency is defined by the transition from low-frequency hearing loss to high-frequency normal hearing, and corresponds to the frequency where the impedance in the dehiscent canal is equal to the cochlear impedance. Above the corner frequency, the SSCD impedance is higher than the cochlear impedance, which effectively stops the shunting of acoustic energy through the canal and leaves air-conducted hearing thresholds unaffected. This corner frequency depends on the location and size of the dehiscence and canal. The predicted bone-conducted audiogram shows low-frequency hypersensitivity that depends on a number of factors: the resonance of the lymph fluids, the middle ear compliance, symmetry in the scala vestibuli and tympani, and symmetry in the round window and middle ear impedances (Figure 2C). These mechanical factors likely explain some of the SSCD patient variability seen with audiometry. Finally, the model has been used to predict some low-frequency mechanics where the SSCD shunts lymph volume velocity (Figure 2D), but the model neglects the effect of traveling waves along the membranous labyrinth that contribute to vestibular biomechanics in SSCD at higher frequencies as described below (34).

Maximal air-bone gap has been correlated with increased dehiscence length in a large multivariate assessment of SSCD patients (50). A study of intracochlear pressures demonstrates that as dehiscence length increases, the pressure drop across the cochlear partition increases, though the effect saturates at about 2–3 mm in length (51). The authors of the study suggest that as the dehiscence length increases, the impedance at the dehiscence is lowered until other limits dominate, and there is little additional decrease in impedance. This length is likely 1–2 times the diameter of the semicircular canal (31, 51).

Middle ear transmission is not responsible for air-bone gap in SSCD patients, evidenced in part by robust click-evoked VEMP responses (22). Other diagnostic tests and middle ear exploration confirm the lack of pathological middle ear conditions in SSCD (35–38, 52–54).

### Vestibular

#### Eye Movements With Sound and Pressure

Sound- or pressure-evoked eye movements generally align with the plane of the dehiscent semicircular canal (55). However, in cases of large dehiscences (≥5 mm) the alignment of the evoked eye movements can be in other planes, thought to occur due to autoplugging of the dura into the superior canal that compresses the membranous duct and reduces canal function (25, 55). MRI imaging has documented the prolapse of middle fossa dura through a superior canal dehiscence and vestibular-ocular reflex testing shows this prevents high-frequency dynamic response within the superior canal (56). Dehiscence size has been shown to affect the frequency that produces the maximal nystagmus response (57). Additionally, some patients exhibit sound-evoked head movements in the same direction as the ocular slow phase (55).

**Figure 3:**
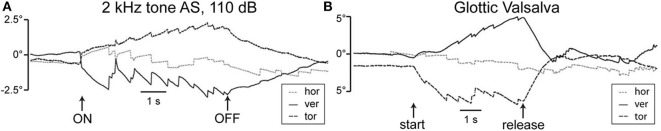
Eye positions recorded from a patient with SSCD. **(A)** Sound-evoked eye movements with 2 kHz tone at 110 dB presented to the left dehiscent ear. Slow phase components are directed upward and clockwise with respect to the patient's point of view, consistent with excitation of the left superior semicircular canal. **(B)** Pressure-evoked eye movements with glottic Valsalva. Slow phase components are principally downward and counterclockwise consistent with inhibition of the left superior semicircular canal. Release causes reversal of the evoked eye movements. Republished from (22), with permission.

In SSCD, eye movements can be evoked by low frequency or static (LF) pressure, or an auditory frequency (AF) stimulus. The biomechanics underlying responses to LF vs. AF stimuli differ. Application of increasing middle ear pressure in response to positive external ear canal pressure or nasal Valsalva maneuver drives slowly increasing deflection of the superior canal cupula in the excitatory ampullofugal direction, while decreased middle ear pressure in response to negative pressure exerted on the external ear canal and increased intracranial pressure in response to glottic Valsalva slowly drives the cupula in the inhibitory ampullopetal direction. Figure 3 demonstrates the slow eye movement with sound (A) or pressure from glottic Valsalva (B). Sound, in contrast, vibrates the cupula leading to excitatory phase-locked canal afferent neuron responses that occur with a short onset latency (34, 58). Sound also triggers wave propagation along the membranous canal that slowly pumps the endolymph in the excitatory or inhibitory direction in a frequency-dependent manner (34). The magnitude and direction of endolymph pumping are highly sensitive to dehiscence location, morphology of the canal, physical properties, and frequency (34) —factors that would be expected to introduce considerable inter-subject variability. Rapid-onset slow-phase eye movements are excitatory, as vibration-evoked phase-locked neural responses evoked by sound are always excitatory (34, 58, 59). This short-latency excitation is superimposed on a slower component arising from endolymph pumping and cupular deflection (33, 34). The short-latency phase-locked responses cease almost immediately upon termination of the sound, whereas long-latency responses slowly return to baseline following the mechanical time constant of the cupula. Therefore, eye movements after cessation of the sound stimulus are a measure of sustained afferent responses to ampullofugal or ampullopetal cupula displacement, while short-latency eye movements near the onset of the sound are a measure of afferent cycle-by-cycle phase-locked responses to cupula vibration. Nonlinear biomechanics underlying these sound-evoked responses is described in more detail in a later section.

#### VEMPs

**Figure 4:**
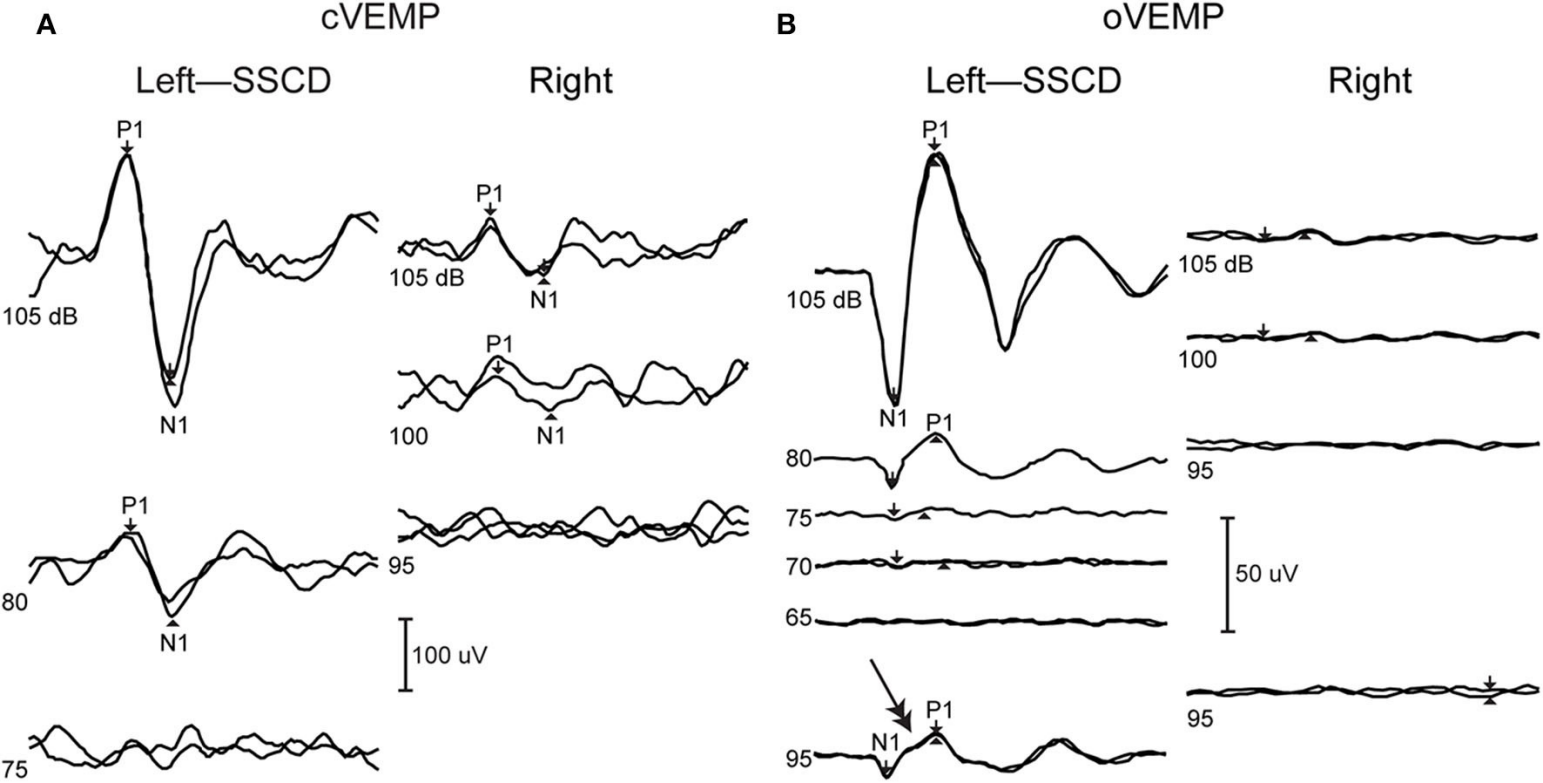
VEMPs from dehiscent (left) and patent (right) ear. **(A)** cVEMP shows increased amplitudes in the dehiscent ear. **(B)** oVEMP shows increased amplitudes as well as abnormal response at super high frequency 4,000 Hz (double arrow). Republished from (70), with permission.

Vestibular Evoked Myogenic Potentials (VEMPs) provide a strong diagnostic indicator of SSCD. The cervical VEMP (cVEMP) pathway is thought to reflect the inhibitory vestibular-colic reflex generated by the activation of saccular macula and potentials are recorded from EMG activity of ipsilateral sternocleidomastoid muscle (60, 61), while the ocular VEMP (oVEMP) is thought to reflect the excitatory vestibular-ocular reflex generated by the activation of utricular macula and responses are recorded from EMG activity of contralateral oblique inferior muscle (62, 63). Both cVEMPs and oVEMPs are diagnostic indicators for SSCD (64), and patients exhibit abnormal, enhanced responses to auditory clicks or tone bursts used in the tests (65). cVEMP amplitudes in the affected labyrinth are increased, and thresholds are lowered (22, 66, 67). oVEMP amplitudes are increased and demonstrate enhanced n10 responses to clicks and 500 Hz tonebursts (68) and 4,000 Hz air-conducted sound or bone-conducted vibration (69). Figure 4 shows typical cVEMP and oVEMP responses from a patient with unilateral SSCD that demonstrate increased amplitudes and an increased oVEMP response to 4,000 Hz (double arrow). It has been shown directly in animal models that creation of a fistula in the superior canal bony labyrinth makes the canal sensitive to auditory frequency sound and vibration (6, 24), which underlies the enhanced oVEMPs in SSCD. The enhanced response has biomechanical origins as described below. After surgical plugging of the dehisced canal, VEMP thresholds and amplitudes normalize (67).

Enhanced activation of the utricle and saccule by sound used in VEMPs testing is explained by the acoustic energy that is shunted away from the cochlea and into conveyed into the vestibular labyrinth. This energy increases the activation of irregularly discharging otolith afferent neurons that are normally activated only at higher stimulus levels (71). When the canal is repaired, the VEMP thresholds normalize as sound energy is no longer being drawn diverted through the vestibule.

VEMP thresholds can be lower in patients with enlarged vestibular aqueduct (67, 72) and/or perilymphatic fistula (73). However, VEMPs have not been found to accurately or substantively diagnose non-SSCD third window syndromes (74).

#### Electrocochleography

**Figure 5:**
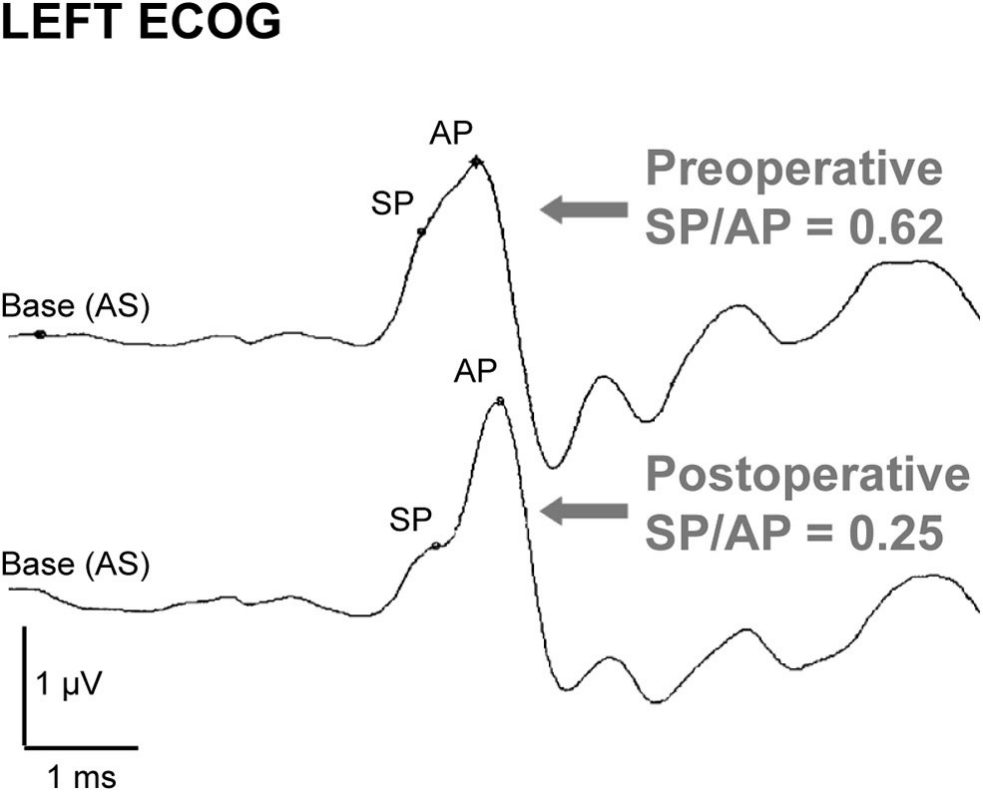
ECoG in a dehiscent ear before and after canal surgery. Preoperative ECoG response shows an elevated SP/AP ratio (>0.4) that normalizes after surgical canal plugging. Republished from (75), with permission. The Creative Commons license does not apply to this content. Use of the material in any format is prohibited without written permission from the publisher, Wolters Kluwer Health, Inc. Please contact permissions@lww.com for further information.

Electrocochleography (ECoG) shows elevated summating potential (SP) relative to the action potential (AP) in the majority of patients with SSCD (SP/AP ratio > 0.4) (75–78). The SP/AP ratio usually normalizes after surgical correction (e.g., Figure 5) and can be monitored intraoperatively to monitor canal occlusion (75, 76), though symptoms can resolve after surgery without normalization of the ratio (78). The SP value is significantly increased in SSCD patients and decreases after plugging (77, 78). The AP value is likely decreased and increases after plugging in most patients (75, 79). However, the decrease in SP amplitude has a greater effect on SP/AP normalization (75). Though not completely understood, the SP is a short-latency stimulus evoked response and the AP a long-latency response. One hypothesis is that the SP response arises in part from high-frequency responses of the vestibular otolith organs that increase with SSCD, and the AP response arises from cochlear responses that decrease with dehiscence (34). The AP would increase after canal plugging due to the acoustic energy being shunted back into the cochlea. Taken together, these two biomechanical factors could explain the change in the SP/AP ratio.

ECoG has been shown to distinguish SSCD patients from normal subjects, though it has not been shown to be reliable for other third window conditions (77). Cochlea-facial nerve dehiscence and third window syndrome patients described by Wackym et al. usually do not have abnormal ECoG data (20). However, an elevated SP/AP ratio (80) and increased SP value (in 4 of 14 patients) (27) has been reported in a few cases of enlarged vestibular aqueduct. In cases of perilymphatic fistula, the SP/AP ratio is elevated in human (81) and an animal model where it normalizes after healing (82). It is hypothesized that SSCD in these cases induces hydrostatic changes similar to those in endolymphatic hydrops, and therefore has a similar effect on ECoG waveform (76). Though these results describe similar results in some other third window conditions, the complexity of different contributions to the ECoG waveform and the variety of these conditions are responsible for the unreliability of this test in identifying other third window conditions.

#### Vestibular Biomechanics

Vestibular symptoms evoked by straining or middle ear pressure arise from the pressure driven fluid flow between the oval window and the dehiscence [shown schematically in Figure 2A, (49)]. Tullio phenomena and sensitivity to auditory frequency sound arise from a more complex biomechanical mechanism. Sound energy that is diverted toward the dehiscence generates a pressure difference across the membranous vestibular labyrinth that can excite traveling waves (33, 34). Lymph fluids are nearly incompressible and inward volume velocity of fluid at the oval window is balanced by outward volume velocity at the dehiscence, plus the outward volume velocity at the round window. The pressure drop in perilymph from the round window to the dehiscence generates a large pressure gradient both along and across the membranous labyrinth between perilymph and endolymph. This large pressure gradient excites propagating waves that originate at the site of the dehiscence and travel along the membranous duct toward the utricle (34). Though the direction of wave propagation from the dehiscence toward the location of sound stimulus might seem counterintuitive, it arises because conservation of fluid mass converts a low-velocity fluid displacement near the relatively large utricular vestibule into a high-velocity fluid displacement near the fistula. As a result, the highest transmembrane pressure gradients occur near the dehiscence, triggering waves that propagate away from the dehiscence (Figure 7).

**Figure 6:**
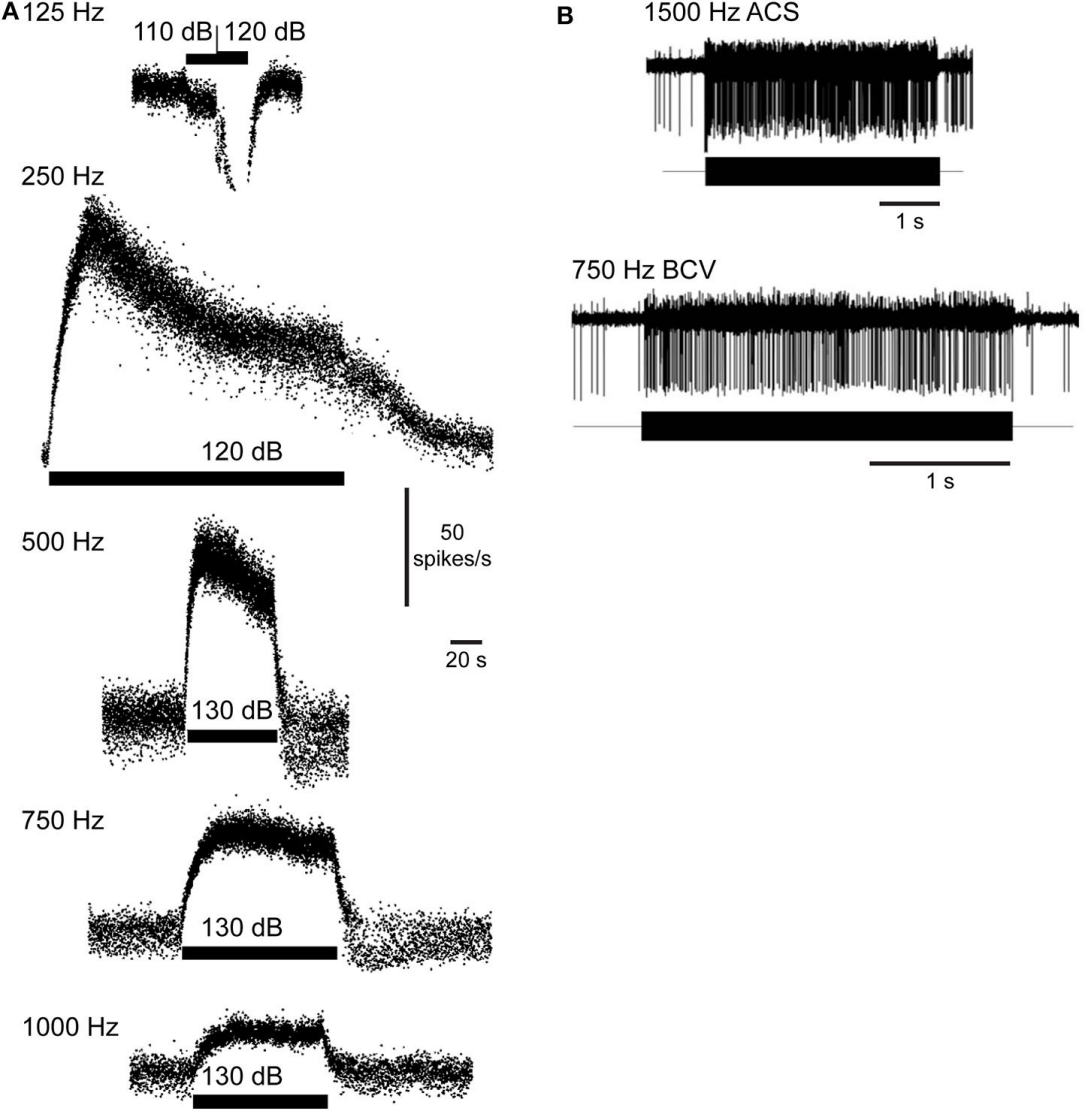
Vestibular afferent neuron responses evoked by fluid vibration and pumping. **(A)** Sustained changes in firing rate in a superior canal afferent neuron after a dehiscence is made in chinchilla superior semicircular canal. Sound evokes a decrease (125 Hz) or increase in afferent firing rate (250, 500, 750, 1,000 Hz). Rise time follows the slow mechanical time constant of the canal. Republished from (83), with permission. **(B)** Phase-locked responses in a superior canal afferent neuron after dehiscence is made in guinea pig superior canal. Sound and bone-conducted vibration at auditory frequencies evoke phase-locking in this irregularly discharging calyx-bearing unit. Republished from (24), with permission. The Creative Commons license does not apply to this content. Use of the material in any format is prohibited without written permission from the publisher, Wolters Kluwer Health, Inc. Please contact permissions@lww.com for further information.

**Figure 7:**
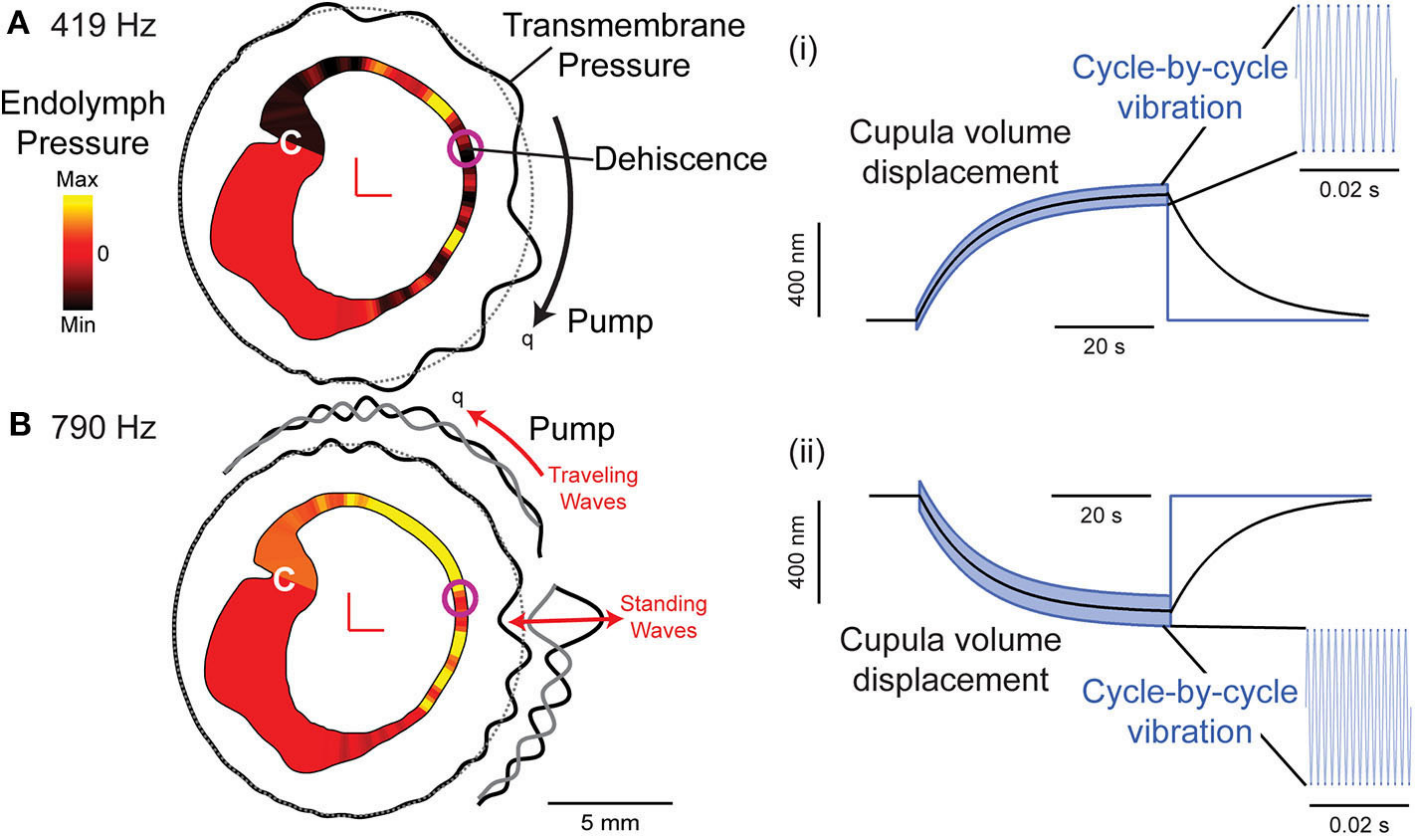
Computational model of a human semicircular canal. **(A,B)** Auditory frequency stimulation at 419 Hz **(A)** and 790 Hz **(B)** evokes slowly developing endolymph pressure distribution (yellow: high; red: zero; and black: low) and a pressure gradient across the cupula (C). Waves travel along the membranous labyrinth away from the site of the dehiscence (transmembrane pressure: black solid line relative to gray dotted line) causing vibration of hair bundles at the stimulus frequency and pumping of endolymph (q) in either direction, ampullofugal for 419 Hz **(A)** and ampullopetal for 790 Hz **(B)**. (i,ii) Cupula displacement where black is the mechanical cupula volume displacement responsible for sustained afferent responses, blue is the cycle-by-cycle cupula vibration responsible for phase-locked afferent responses at 419 Hz (i) and 790 Hz (ii). Based on (34).

AF sound-excited waves in the labyrinth have two effects that are demonstrated in recordings of vestibular afferent neurons. First, the waves passing through the ampulla vibrate sensory hair bundles at the sound frequency. Irregularly discharging afferent neurons respond to this auditory-frequency vibration by firing phase-locked action potentials (Figure 6B). Second, traveling waves in the membrane interact nonlinearly with the lymph fluids to pump endolymph. Traveling waves are generated on both sides of the dehiscence, but reflections cause one wave to dominate and generate net endolymph flow predominantly in the ampullofugal or ampullopetal direction in a frequency-dependent manner (Figure 7). Canal asymmetry is necessary to observe net endolymph pumping. Regularly discharging afferent neurons respond to cupula deflection caused by endolymph pumping by increasing or decreasing their action potential firing rate with a build-up rate that follows the slow mechanical time constant of canal macromechanics (Figure 6A).

Phase-locked responses are lost after plugging the canal (24). A biomechanical model predicts that sound-evoked vibration and endolymph pumping is present in normal canals, but is very small and insufficient to evoke neural responses (84), except at very high sound pressure levels (85).

#### Repair and Plugging

Patients with mild symptoms can reduce exposure to loud sounds and avoid physical straining, and those with pressure sensitivity can benefit from a tympanostomy tube (22). Patients with debilitating symptoms are candidates for surgical repair, and about one-third elect to have surgery (70). Canal plugging achieves long-term control more often than resurfacing and is usually the procedure of choice (22). Patients typically see long-term improvement after canal plugging in symptoms such as sound- or pressure-evoked vertigo (86), autophony (87), dizziness handicap (88), and health-related quality of life (89). Balance measures are impaired immediately after surgical repair (90), but partially recover after 6 weeks to the extent offered by central compensation (91). Compensatory vestibular-ocular reflexes (86) and dynamic visual acuity (92) do not fully recover. Vestibular physical therapy is useful in the postoperative period to aid in recovery (91, 93). In animal models, canal plugging impairs the low frequency VOR and profoundly reduces single unit afferent sensitivity to low-frequency head rotations (>100 fold), but introduces only modest attenuation for high-frequency head rotations (>10 Hz) (94–96). The residual sensitivity at high-frequencies arises from acceleration-induced transmembrane fluid pressure that deforms the labyrinth and deflects the cupula (84). Observations in animal models are consistent with vestibulo-ocular reflexes (VOR) measured postoperatively in patients in that compensatory eye movements are present in response to rotary head thrusts but compromised relative to controls (86, 92, 97, 98). The reduced VOR following surgical plugging putatively reflects broad-band attenuation of sensitivity caused by the procedure, while persistence of a partial VOR reflects residual sensitivity to high-frequency angular head movements. As an alternative to canal plugging, round window reinforcement has been shown to reduce most symptoms in most patients with intractable superior semicircular canal dehiscence with the exception of hearing loss (99).

Mild high-frequency sensorineural hearing loss occurs in ~25% of patients (100) though significant hearing loss is rare (21). New-onset benign paroxysmal positional vertigo has been reported in up to 25% of postoperative patients likely due to otoconia or plugging material that becomes mobilized in the endolymph (101). Revision surgery is sometimes necessary when symptoms do not cease or reoccur and, in one report, is performed in approximately 10% of cases, though revisions are reported to carry a lower rate of success than primary surgery (102).

## Conclusion

Superior semicircular canal dehiscence is the most common third-window syndrome. Patients present with sound- or pressure-evoked eye movements and dizziness, decrease in air-conducted hearing, and increase in bone-conducted hearing. The biomechanics of this disorder involves a shunting of acoustic energy away from the cochlea and toward the dehiscent semicircular canal. This increases sound-evoked VEMPs responses, and causes an increase in the audiometric air-bone gap. ECoG tests are consistent with an increase in the short-latency response from the vestibular organs relative to the long-latency response from the cochlea. Various other third window conditions have similar presentations. A dehiscence or fistula located in the bony canal renders the canal sensitive to AF sound and LF pressure. LF responses reflect slow displacements of the cupula in the excitatory or inhibitory direction driven by pressure-evoked deformation of the labyrinth. The specific afferent neurons most sensitive to LF cupula displacements fire action potentials with regularly spaced inter-spike intervals—neurons that provide sustained inputs to the central nervous system. In contrast, AF sound evokes waves that travel along the membranous labyrinth emanating from the site of the dehiscence. The waves vibrate the hair bundles leading to short-latency excitatory phase-locked neuron responses. The specific afferent neurons that are most sensitive to AF vibration fire action potentials with irregularly spaced inter-spike intervals—neurons that provide transient inputs to the central nervous system. These AF sensitive afferent neurons drive short-latency sound-evoked nystagmus in third window patients. In addition, sound generates a slow displacement of the cupula through wave-driven endolymph pumping. This can excite or inhibit regularly discharging afferents, depending on the subject-specific morphology and stimulus frequency, driving a long-latency component that superimposes on top of the short-latency sound-evoked nystagmus. Canal plugging, if complete, removes the third window and eliminates the syndrome.

## Author Contributions

MI and RR drafted manuscript, edited and revised manuscript, and approved final version of manuscript. MI prepared figures. All authors contributed to the article and approved the submitted version.

## Conflict of Interest

The authors declare that the research was conducted in the absence of any commercial or financial relationships that could be construed as a potential conflict of interest.
